# Comprehensive Insights into Keloid Pathogenesis and Advanced Therapeutic Strategies

**DOI:** 10.3390/ijms25168776

**Published:** 2024-08-12

**Authors:** Hyun Jee Kim, Yeong Ho Kim

**Affiliations:** 1 Department of Dermatology, International St. Mary’s Hospital, College of Medicine, Catholic Kwandong University, Incheon 22711, Republic of Korea; hyunjee0921@hanmail.net; 2 Department of Dermatology, Seoul St. Mary’s Hospital, College of Medicine, The Catholic University of Korea, Seoul 06591, Republic of Korea

**Keywords:** keloid, immunology, therapeutic targets

## Abstract

Keloid scars, characterized by abnormal fibroproliferation and excessive extracellular matrix (ECM) production that extends beyond the original wound, often cause pruritus, pain, and hyperpigmentation, significantly impacting the quality of life. Keloid pathogenesis is multifactorial, involving genetic predisposition, immune response dysregulation, and aberrant wound-healing processes. Central molecular pathways such as TGF-β/Smad and JAK/STAT are important in keloid formation by sustaining fibroblast activation and ECM deposition. Conventional treatments, including surgical excision, radiation, laser therapies, and intralesional injections, yield variable success but are limited by high recurrence rates and potential adverse effects. Emerging therapies targeting specific immune pathways, small molecule inhibitors, RNA interference, and mesenchymal stem cells show promise in disrupting the underlying mechanisms of keloid pathogenesis, potentially offering more effective and lasting treatment outcomes. Despite advancements, further research is essential to fully elucidate the precise mechanisms of keloid formation and to develop targeted therapies. Ongoing clinical trials and research efforts are vital for translating these scientific insights into practical treatments that can markedly enhance the quality of life for individuals affected by keloid scars.

## 1. Introduction

A keloid is a type of pathological scarring characterized by excessive growth of fibrous tissue extending beyond the original wound boundaries. These scars are disfiguring and can cause pain, itching, and hyperpigmentation, leading to significant physical and mental distress. Keloid formation is a clinical challenge due to its unpredictable nature and high recurrence rate even after treatment.

Epidemiological studies have revealed significant disparities in keloid prevalence among different racial groups, suggesting a strong genetic influence. Individuals with darker skin tones, such as those of African and Asian descent, are at a higher risk [1,2]. This is evidenced by the prevalence rate of 2.4% in Black populations, 1.1% in Asians, and 0.4% in Caucasians [2]. Moreover, global keloid prevalence varies drastically, ranging from 0.09% in the United Kingdom to 16% in Congo, further underscoring the impact of ethnicity [3].

The familial occurrence of keloids also supports the role of genetic predisposition. Studies have shown a higher incidence of keloids among family members of affected individuals [4,5,6]. For instance, the prevalence rate of keloid in the first, second, and third-degree relatives of Chinese keloid patients was 7.62%, 0.38%, and 0.035%, respectively [7].

Age is another factor influencing keloid susceptibility, with the highest incidence occurring between the ages of 10 and 30 years [8]. Gender also plays a role, with keloids being more common in females, although the reasons for this remain unclear [1,2].

Apart from these factors, systemic conditions like hypertension, vitamin D deficiency, and atopic dermatitis have been linked to an increased risk of keloid formation, suggesting that systemic health may play a role in scar formation [9,10,11,12,13,14,15].

The pathogenesis of keloids is complex and multifactorial, involving genetic susceptibility, immune response dysregulation, and aberrant wound-healing processes. Despite extensive research, the exact mechanisms remain poorly understood, highlighting the need for further investigation and the development of more effective treatment strategies.

## 2. Pathogenesis of Keloid Formation

**Figure 1:**
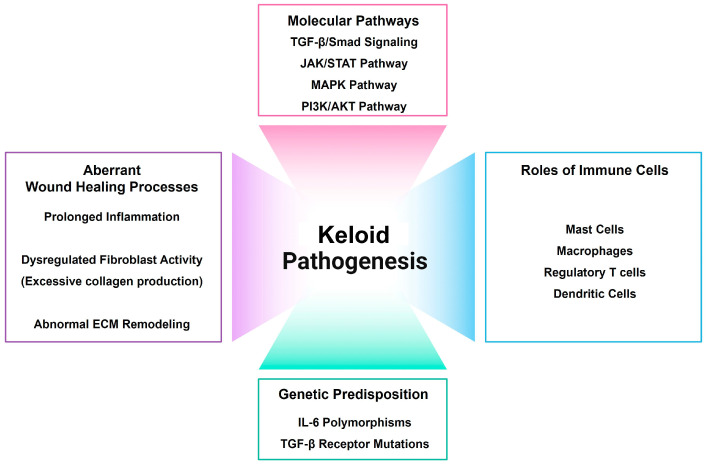
Keloid pathogenesis.

The pathogenesis of keloid formation involves a multifaceted interplay of genetic, immunological, and mechanical factors that disrupt normal wound healing, leading to persistent fibroblast activation and excessive extracellular matrix production (Figure 1) [16,17,18,19,20,21,22,23,24,25,26,27,28,29,30,31,32]. 

### 2.1. Dysregulation of Wound-Healing Process

Normal wound healing involves hemostasis, inflammation, proliferation, and remodeling [33]. Dysregulation at any of these stages can lead to pathological scarring, with the remodeling phase being particularly critical in keloid formation. The abnormal wound-healing process in keloids is characterized by persistent inflammation, excessive fibroblast activation, and an imbalance between collagen synthesis and degradation [34,35,36,37]. 

During the inflammatory and proliferative phases, immune cells such as mast cells, macrophages, and lymphocytes infiltrate the wound site, playing roles in modulating the healing process [33]. Mast cells, for instance, interact with fibroblasts to enhance collagen production through pathways such as PI3K/Akt/mTOR and TGF-β1/Smad, contributing to the persistent fibroblast activation seen in keloids [38,39,40,41,42]. Macrophages, particularly the M2 subtype, further promote fibroblast proliferation and ECM deposition by secreting cytokines like TGF-β and PDGF [43,44,45]. 

The remodeling phase is where the critical pathological features of keloid formation become evident. This phase typically involves the replacement of collagen III with collagen I and the regression of blood vessels to form a mature scar [35,46]. However, in keloids, this phase is marked by abnormal collagen metabolism, where an imbalance between collagen synthesis and degradation leads to excessive accumulation of disorganized type I and III collagen [47,48]. This dysregulation is partly due to the altered expression of matrix metalloproteinases (MMPs) and their inhibitors, known as tissue inhibitors of metalloproteinases (TIMPs) [49]. In keloids, levels of MMP-1, MMP-2, MMP-13, TIMP-1, and TIMP-2 are increased, while MMP-3 levels are reduced compared to normal scars [50,51]. These changes contribute to the excessive collagen deposition and increased type I/III collagen ratio observed in keloid tissue [52]. This pathological remodeling process leads to the dense, fibrotic tissue that is characteristic of keloids.

### 2.2. Genetic Predisposition

Familial cases of keloids suggest a genetic predisposition [4,5,6]. Several immune pathway-associated susceptible genotypes have been identified, including polymorphisms of interleukin (IL)-6 and transforming growth factor (TGF)-β receptors [21,22,23,53,54,55]. Genetic studies have identified polymorphisms in several single nucleotides that are associated with keloid formation [27,56]. These genetic factors may contribute to individual susceptibility to keloid formation.

### 2.3. Molecular Pathways

Dysregulated molecular pathways, particularly the TGF-β/Smad signaling pathway, play a central role in keloid pathogenesis. Other pathways, including JAK/STAT, MAPK, PI3K/AKT, and mechanical transduction pathways (integrin, YAP/TAZ), also contribute to the abnormal behavior of keloid fibroblasts.

#### 2.3.1. TGF-β/Smad Signaling Pathway

TGF-β1 is a multifunctional cytokine implicated in keloid pathogenesis due to its role as a key regulator of fibrogenesis [57]. TGF-β1 has a pro-fibrotic effect by enhancing human fibroblast cell proliferation, increasing collagen synthesis, and reducing collagen degradation [58]. It maintains the ECM by inducing growth factors like connective tissue growth factor (CTGF) and vascular endothelial growth factor (VEGF) and contributes to chronic inflammation by downregulating dipeptidyl peptidase-4 (DPP4) expression [59]. Keloid fibroblasts are particularly sensitive to TGF-β, showing resistance to apoptosis and increased cell rigidity through the expression of smooth muscle actin (SMA) [60,61]. TGF-β1 also increases the expression of C-MYC and its downstream splicing regulator, polypyrimidine tract-binding protein, in keloid fibroblasts, which is a key factor in tumorous growth [62].

The canonical TGF-β/Smad pathway begins with the binding of TGF-β to a complex of serine/threonine kinase receptors on the cell surface, composed of type I and type II receptors [63]. Upon ligand binding, the type II receptor phosphorylates the type I receptor, which then phosphorylates receptor-regulated Smads (R-Smads), specifically Smad2 and Smad3 [63]. Phosphorylated Smad2 and Smad3 form a complex with the common mediator, Smad4 (Co-Smad) [63]. This Smad complex translocates to the nucleus, where it regulates the transcription of target genes involved in ECM production and fibrosis, including genes encoding for collagen and connective tissue growth factors [64]. Smad7 acts as an inhibitory Smad, providing negative feedback by preventing the association of R-Smads with the TGF-β type I receptor and facilitating their degradation via ubiquitination [65,66,67].

In keloids, several upstream modulators of the TGF-β1/Smad pathway, including activating transcription factor 3 (ATF3), CR6-interacting factor 1 (Crif1), NLR family CARD domain containing 5 (NLRC5), and nuclear receptor subfamily 3, group C, member 1 (NR3C1), are overexpressed in keloid fibroblasts, enhancing the TGF-β1/Smad pathway [68,69,70]. Additionally, hypoxia-inducible factor-1α (HIF-1α) and high-temperature requirement factor A1 (HTRA1) activate the TGF-β1/Smad pathway and promote keloid formation [71,72].

MicroRNAs (miRNAs) and long non-coding RNAs (lncRNAs) are capable of modulating TGF-β/Smad signaling. For instance, pro-fibrotic miR-21, which is upregulated in keloids, enhances the TGF-β/Smad pathway by downregulating Smad7, thus promoting fibrosis [73]. Conversely, anti-fibrotic miRNAs are typically downregulated in keloid fibroblasts [74,75,76,77]. Several molecules like BMP and activin membrane-bound inhibitor (Bambi), Dickkopf-3 (DKK3), and the receptor for activated C-kinase 1 (RACK1) can attenuate TGF-β1-induced fibrosis but are downregulated in keloid fibroblasts [78,79,80]. IL-37, an inhibitor of inflammation and TGF-β regulator, is found at lower levels in more severe keloids [81,82,83].

Strategies targeting the TGF-β/Smad pathway have shown promise in reducing keloid fibroblast activity. For example, overexpression of inhibitory factors like Smad7 or using molecules that interfere with Smad2/3 phosphorylation can attenuate the fibrotic process [84]. Moreover, compounds that inhibit upstream activators of the pathway or modulate miRNA expression are being explored as potential treatments [33].

#### 2.3.2. JAK/STAT Pathway

The Janus kinase (JAK)-signal transducer and activator of transcription (STAT) pathway is a key signaling pathway activated in response to cytokines, which are small proteins involved in cell signaling. This pathway plays a crucial role in regulating various cellular responses, including proliferation, differentiation, migration, apoptosis, and cell survival [85]. In the context of keloids, the JAK-STAT pathway is activated by pro-inflammatory factors overexpressed in keloid tissues, such as IL-1β, IL-6, IL-17, and tumor necrosis factor (TNF)-α [86,87,88,89]. These cytokines trigger a cascade of events within the JAK-STAT pathway, ultimately leading to the dysregulated cellular responses observed in keloids.

Research has identified STAT3 signaling as the most significantly enriched gene ontology term in keloid fibroblasts (KFs), the primary cells responsible for collagen production in keloids [90]. STAT3, a protein within the STAT family, is activated through phosphorylation, a process enhanced in both keloid tissue and KFs [91]. This activation suggests that STAT3 plays a crucial role in keloid pathogenesis, potentially driving the excessive collagen production and proliferation characteristic of keloids.

Interleukins (ILs) are a group of cytokines that play a significant role in keloid formation. IL-6 and IL-17, in particular, have been shown to elicit STAT3 phosphorylation in both healthy and keloid fibroblasts [29,88,89,92,93]. Notably, IL-6 has been identified as the primary cytokine responsible for triggering STAT3 phosphorylation in these cells [92]. This finding suggests that targeting IL-6 or its receptor could be a potential therapeutic strategy for keloids, as it may disrupt the JAK-STAT pathway and its downstream effects on cell proliferation and collagen synthesis.

Other interleukins, such as IL-1β and IL-10, have also been implicated in keloid pathogenesis through their interactions with the JAK-STAT pathway. IL-1β enhances fibrosis in the late stage of wound healing, and blocking it has been shown to inhibit keloid progression [94]. Conversely, IL-10 expression is decreased in keloids, and its overexpression has been found to decrease inflammation and promote regenerative healing [95,96]. These findings suggest that modulating the levels or activity of these interleukins could be a potential avenue for keloid treatment.

The JAK-STAT pathway influences several growth factors involved in keloid pathogenesis. JAK2 inhibition reduces the expression of connective tissue growth factor (CTGF), even in the presence of TGF-β stimulation, highlighting the pathway’s role in fibrosis [97]. Additionally, STAT inhibition decreases the mRNA expression of vascular endothelial growth factor (VEGF) in keloid fibroblasts, suggesting a role in angiogenesis regulation [97].

#### 2.3.3. MAPK Pathway

The mitogen-activated protein kinase (MAPK) pathway, a complex network of extracellular signal-regulated kinase (ERK), c-Jun N-terminal kinase (JNK), and p38 kinase plays a multifaceted role in keloid pathogenesis [98]. Primarily, it crosstalks with the TGF-β1/Smad signaling pathway [99]. TGF-β1, overexpressed in KFs, activates the MAPK pathway, influencing Smad protein phosphorylation and activation of genes like plasminogen activator inhibitor (PAI)-1, which promotes collagen accumulation [100].

Within the MAPK pathway, ERK1/2 is reportedly significantly activated in KFs, suggesting a potential contribution to their excessive proliferation, migration, and ECM synthesis [101]. ERK inhibitors, like FR180204, can counteract these effects [101]. The p38 MAPK pathway is activated by TGF-β1 and IGF-1, leading to hyperproliferative, migratory, and anti-apoptotic behavior of KFs [102]. TNF-α further amplifies this effect via p38 MAPK [103]. Notably, drugs like thalidomide and nintedanib, targeting p38 MAPK, have shown promise in limiting KF function and keloid growth [104,105].

Osteomodulin (OMD), a protein highly expressed in keloid tissues, has been shown to activate p38 MAPK, potentially enhancing the tumor-like characteristics of KFs [106]. Silencing OMD inhibits these effects [106]. Additionally, protein tyrosine phosphatase 1B (PTP1B), downregulated in keloids, has been suggested to act via the MAPK/ERK pathway to control KF activity [101]. Its overexpression has been shown to suppress KF function, suggesting a potential therapeutic avenue [101].

Further, proenkephalin (PENK) from placental mesenchymal stem cells suppresses p38 MAPK signaling in KFs, reducing proliferation and migration, and promoting apoptosis, suggesting another therapeutic target [107]. 2-Methoxyestradiol (2ME2), targeting p38 within the MAPK/ERK pathway, has been found to inhibit KF proliferation, further highlighting the potential therapeutic potential of manipulating MAPK signaling in keloid treatment [108].

#### 2.3.4. PI3K/AKT Pathway

The phosphoinositide 3-kinase (PI3K)/AKT signaling pathway plays a role in regulating fibroblast proliferation, migration, and differentiation into myofibroblasts [109]. Analysis has revealed that differentially expressed genes in keloid tissues are primarily associated with the PI3K/AKT signaling pathway [110].

Studies have shown that the knockdown of Runt-related transcription factor 2 (Runx2) and circCOL5A1 suppressed KF proliferation and migration while promoting KF apoptosis by suppressing the PI3K/AKT pathway [110,111]. Additionally, other factors like CD26 upregulate the proliferation and invasion of KFs through the IGF-1-induced PI3K/AKT/mTOR pathway [112].

Long non-coding RNAs (lncRNAs) are also implicated in the activation of the PI3K/AKT pathway in keloids. Specifically, uc003jox.1 is a lncRNA upregulated in keloid tissues that promote KF proliferation and invasion through this pathway [113]. Another study showed that knocking down uc003jox.1 leads to decreased phosphorylation of PI3K and AKT, thereby reducing the activity of the pathway and inhibiting cell proliferation and invasion [113].

From a therapeutic perspective, targeting the PI3K/AKT pathway with inhibitors like CUDC-907, sunitinib, or wubeizi (*Rhus chinensis* Mill.) ointment has shown promise in preclinical models, suggesting its potential as a therapeutic strategy for keloid treatment [114,115,116].

#### 2.3.5. Mechanical Transduction Pathways

Mechanical stimulation, such as skin tension and stretching, plays a role in keloid formation. It leads to excessive proliferation of wound fibroblasts, ECM deposition, and secretion of pro-fibrotic factors, which in turn increase the stiffness of the keloid matrix [18]. The increased matrix stiffness further activates the fibrotic phenotype of keloid fibroblasts, creating a continuous loop that invades surrounding normal tissue [18]. Several mechanotransduction pathways translate mechanical stimuli into biochemical signals, driving the cellular behaviors that contribute to keloid pathogenesis.

The TGF-β/Smad signaling pathway is one such pathway. Mechanical forces enhance TGF-β activation [117]. Upon binding to its receptors, TGF-β activates Smad proteins, which translocate to the nucleus and regulate fibrosis-related genes [12,33].

The integrin signaling pathway is also involved. Mechanical forces activate integrins, converting mechanical signals into chemical signals that promote the proliferation and differentiation of fibroblasts and the secretion of collagen [118]. Integrins also play a role in TGF-β activation, further contributing to keloid progression [119]. The FAK/Erk pathway is involved in integrin-induced gene expression [18].

The YAP/TAZ signaling pathway is another key mechanosensor. YAP (Yes-associated protein) and TAZ (transcriptional coactivator with PDZ-binding motif) respond to a wide range of mechanical cues, such as ECM stiffness and shear stress [120]. Increased matrix stiffness in keloids activates YAP/TAZ, leading to the expression of pro-fibrotic genes, increased α-SMA expression, and excessive matrix deposition [121,122].

Calcium ion signaling is also involved. The Gq-coupled receptor, activated by stretch stimulation, can activate PLCβ and produce DAG and IP3 [123]. IP3 acts on the intracellular calcium pool to release Ca^2+^, resulting in increased intracellular free calcium ion concentration and the subsequent intracellular reaction of the Raf/MEK/Erk pathway [124]. Mechanical stimuli also increase Piezo1 channel expression, which is a mechanically activated channel (MAC) and promotes fibroblast proliferation, migration, and differentiation [125,126].

## 3. Established Treatments for Keloids

Established treatments for keloids include silicone dressings, topical corticosteroids, cryotherapy, surgical options, radiation therapy, laser therapies, and various intralesional injections (triamcinolone, 5-fluorouracil, verapamil, and botulinum toxin A). These treatments aim to reduce scar size and symptoms but are associated with variable success rates and potential side effects (Table 1).

### 3.1. Silicone Dressings

Silicone dressings (silicone gel sheets, silicone gels) are a non-invasive treatment option for the prevention and treatment of keloid scars. This treatment modality is considered safe and effective, gradually improving the color, size, erythema, pliability, pain, and pruritus of scars [127]. Silicone dressings work primarily through skin occlusion and hydration rather than direct anti-scarring effects. The mechanism of action of silicone dressings is due to the hydration of the stratum corneum and the modulation of cell signaling between fibroblasts and keratinocytes, which is mediated by cytokines [127].

Silicone gel sheets showed statistically significant reductions in scar thickness and color improvement compared to no treatment [128]. Silicone gel was later developed to treat scars on areas where it is difficult to fix a silicone sheet, such as the scalp or joints, or on the face where silicone dressing is not cosmetically desirable [129]. Significant scar improvement was observed following the twice-daily application of silicone gel for two months after surgical procedures [130].

### 3.2. Topical Corticosteroids

Topical corticosteroids, including creams, ointments, and lotions, are commonly used in clinical practice for managing keloid scars. Corticosteroids prevent and treat keloids through anti-inflammatory, immunomodulatory, anti-fibroblast proliferation, anti-angiogenesis, and ECM remodeling effects [131]. They bind to glucocorticoid receptors, regulating gene expression and reducing pro-inflammatory cytokine production [132,133,134,135,136,137,138]. Corticosteroids suppress T cell activation, inhibit Th1 and Th17 responses, and promote Th2 and regulatory T cells [139,140]. They reduce fibroblast proliferation by halting the cell cycle and inducing apoptosis [141,142]. Additionally, corticosteroids decrease VEGF mRNA, reduce angiogenesis, and regulate TGF-β1 and bFGF to suppress collagen synthesis while enhancing collagen degradation [141,143,144,145,146,147,148]. These mechanisms make them effective in treating and preventing scars.

Multiple daily applications of corticosteroid cream have demonstrated excellent outcomes in treating existing keloids, as shown in case series studies [238]. Also, the pruritus and pain symptoms of keloids were notably relieved with treatment with TAC lotion [239]. Another study combining TAC injections (once every 2 weeks, five times in total) and corticosteroid ointments (twice a day, 6 months in total) showed reduced recurrence rates of 14.3% for keloids when applied postoperatively [240]. While effective, topical corticosteroids have limited transdermal efficiency due to their short action time, requiring frequent application and occlusion for optimal results [131]. Additionally, continuous use increases the risk of adverse reactions like skin atrophy, telangiectasia, and depigmentation [131].

### 3.3. Cryotherapy

Intralesional (IL) cryotherapy for keloid scar treatment involves freezing the scar tissue from the inside, aiming to reduce keloid scar volume and alleviate pain and pruritus [149].

The mechanism of action involves two phases: a physical phase where rapid freezing causes direct cell injury and a vascular phase where microcirculation damage leads to ischemic necrosis, further destroying the scar tissue [150,151]. Cryotherapy also prevents keloid recurrence by inducing the differentiation of abnormal fibroblasts into a normal phenotype and preventing wound contraction [152,153,154,155,156,157].

A systematic review of eight studies found that IL cryotherapy reduces keloid scar volume by an average of 51% to 63%, with scar recurrence rates ranging from 0% to 24% [149]. However, complete scar eradication was not achieved on average [149]. Hypopigmentation was predominantly observed in patients with Fitzpatrick skin types IV–VI [149].

IL cryotherapy could potentially complement existing treatments, particularly in cases where non-surgical options have failed, as combination therapy, or as an alternative to excision with adjuvant radiation when radiotherapy is unsuitable [149].

### 3.4. Surgical Options

Small keloids can be radically resected, while larger or more numerous keloids may benefit from partial or core excision to reduce their size or quantity [158]. However, radical or complete keloid resection can stimulate collagen synthesis, increasing the risk of recurrence and potentially leading to a larger keloid than the original [159]. Therefore, intramarginal (core) excision is sometimes preferred to minimize this risk [160,161,162,163]. Regardless of the excision technique, adjuvant therapies like radiation therapy should always follow surgery to minimize recurrence rates [164]. Additionally, surgical techniques aimed at reducing tension, such as subcutaneous and deep fascial tensile-reduction sutures, Z-plasties, and local flaps, can further decrease the likelihood of recurrence [165,166,167,168,169,170,171].

Z-plasties, combined with excision, tension-reducing sutures, and radiotherapy, have shown promising results in reducing keloid recurrence in the chest and upper arms [168,170].

For large keloids, flaps are preferred over skin grafts as they allow for post-operative expansion and reduce the risk of scarring [158]. However, donor sites require careful management to prevent new keloids [171].

Ear keloids have specific recommendations: wedge excision for earlobe keloids and core excision for auricular cartilage keloids [172]. Both techniques, when combined with additional therapies like radiotherapy or steroid injections, have demonstrated good outcomes in minimizing recurrence [169,173,174].

### 3.5. Radiation Therapy

Radiation therapy is a common adjuvant therapy after keloid excision. Post-surgical radiotherapy for keloid management acts by slowing down angiogenesis and proliferation of new fibroblasts [175]. This effect is particularly potent during the early phases of wound healing when developing endothelial vascular beds, which are highly radiosensitive [176]. Therefore, studies recommend starting radiation therapy within 72 h of surgical excision [177,178].

There are several radiotherapy techniques available. While brachytherapy has shown superior local control compared to X-rays in one meta-analysis, its invasiveness and limited availability are disadvantages [179,180]. External beam radiotherapy using electrons or photons is a good alternative due to its accessibility, feasibility, and non-invasive nature [180]. Hypofractionated radiotherapy with a BED of more than 28 Gy (α/β value of 10) within 24 h after excision is recommended [180].

Numerous studies confirm the effectiveness of combining surgical excision with adjuvant radiotherapy, reporting local control rates between 67% to 98% and significantly lower recurrence rates (<20%) compared to primary radiotherapy alone [179,181,182,183,184].

Radiotherapy for keloid treatment is generally well-tolerated, with side effects such as erythema, desquamation, and pigmentation changes being mild and transient [185,186]. While there’s concern about secondary malignancies after keloid radiotherapy, the risk is considered very low. Ogawa et al. found five cases of radiation-induced tumors in keloid patients, but the relationship with radiotherapy was unclear [182]. Other studies have found no association between secondary malignancy and keloid radiotherapy [179,183,186,187,188].

### 3.6. Laser Therapies

Laser therapies are a widely used modality for the treatment of keloid scars, utilizing high-energy light to selectively target and remodel abnormal collagen while minimizing damage to surrounding healthy tissue [189,190,191]. This approach effectively reduces scar size, thickness, and redness [189,192]. Various laser modalities are employed, each with unique mechanisms and advantages.

Ablative CO_2_ lasers remove the top skin layer, stimulating new collagen growth and effectively treating deeper scars, but with the potential for longer recovery times [189]. PDL targets blood vessels, reducing redness and inflammation in superficial scars, often requiring multiple sessions [193]. Fractional lasers, including fractional CO_2_ (FCO_2_) and Er:YAG, create microscopic thermal zones that induce controlled injury and promote skin remodeling, effectively treating both superficial and deep scars with minimal downtime [192,193].

Fractional ablative lasers like CO_2_ and Er:YAG are effective in improving excessive scars, with FCO_2_ alone showing significant results [189,194]. A network meta-analysis of 18 studies (550 patients) revealed that FCO_2_ plus 5-FU was most effective in reducing scar severity (Vancouver Scar Scale) and thickness compared to other interventions and controls [195]. However, it showed no significant effect on erythema, vascularity, or pigmentation [195]. FCO_2_ plus CO_2_ was most effective for pliability improvement [195].

Pulsed dye laser (PDL) is another commonly used laser for scar treatment. While it may not be as effective as FCO_2_ plus 5-FU in reducing scar thickness and improving pliability, some studies suggest its potential to reduce erythema when combined with TAC and 5-FU [193].

Laser treatments are generally well-tolerated but can cause side effects such as pain, hyperpigmentation, hypopigmentation, scarring, and infection [190,191].

### 3.7. Intralesional Injections

Intralesional injections provide a direct method to deliver therapeutic agents into the keloid scar tissue. This approach aims to reduce the size, symptoms, and recurrence of keloids by targeting the underlying pathological processes.

Intralesional corticosteroids, such as triamcinolone acetonide (TAC), are among the most commonly used agents for this purpose. When used immediately after surgery, it reduces the likelihood of keloid recurrence by 50% to 91.90% within 5 weeks post-surgery [196,197,198]. TAC induces a specific protein involved in the System A amino acid transport in human keloid fibroblasts, leading to reduced collagen production and inhibition of alpha-2-macroglobulin, which subsequently inhibits collagenase. Injections should be administered in the papillary dermis, where collagenase is produced, rather than in the subcutaneous tissue to avoid causing underlying fat atrophy [199,200]. For the appropriate dosage per cm^2^ of the injection, 1–10 mg of TAC, depending on the size of the lesion, is recommended to be administered at four-week intervals [201]. It is crucial to monitor patients for potential side effects, such as skin atrophy and hypopigmentation, which remain for between six and twelve months [200,202]. The combination of TAC with cryotherapy appears to be more effective, or at least comparable, to other combinations such as 5-fluorouracil or the 585 nm flashlamp-pumped pulsed-dye laser [203,204,205].

In addition to corticosteroids, other agents are also used for intralesional injections. For instance, 5-fluorouracil (5-FU) is a chemotherapeutic agent that has shown effectiveness in keloid treatment by inhibiting fibroblast activities such as proliferation, invasion, and matrix production and inducing endothelial cell apoptosis and destroying neovascular structures in keloids [206,207,208,209,210]. When combined with corticosteroids, 5-FU can enhance therapeutic outcomes [211,212,213,214,215]. In combination therapy, the most commonly used ratios of 5-FU and TAC are 1:9 or 1:3, with protocols displaying considerable variation in session intervals, ranging from once a week to every four weeks [216].

Bleomycin, an antitumor drug that disrupts DNA synthesis in proliferating cells, is another agent used in intralesional injections [217]. Its effectiveness in treating keloids is attributed to its inhibition of lysyl oxidase, an enzyme crucial for collagen maturation and TGF-β1 expression, thereby reducing collagen production and fibroblast activity [218]. Additionally, bleomycin may inhibit collagen synthesis by fibroblasts and induce fibroblast apoptosis [219,220,221]. Intralesional injections of bleomycin for keloids are well-tolerated, with minimal local and systemic side effects, indicating that this treatment can be considered as a first-line therapy for keloids [222]. However, intralesional bleomycin is painful, which can be alleviated by injecting triamcinolone acetonide immediately afterward, as the pain was reduced in minutes or even seconds, suggesting that this combination could be beneficial [221]. Regarding the bleomycin dose, 0.4 mL/cm^2^ (equivalent to 0.60 IU/cm^2^) was applied following a local anesthetic in a previous study using the dermojet, but a recent study suggested applying 0.375 IU of bleomycin per 1 cm^2^ using insulin syringes, injecting the bleomycin while slowly withdrawing the 1 cm-long needle [221,223]. It is simpler to inject 0.25 mL with 0.5 mL or 1 mL insulin syringes [221]. The maximum volume per session is 2 mL (3 IU), which is sufficient to treat eight keloids of 1 cm^2^ or eight quadrants in larger keloids [221]. This dosage is considered effective and safe regardless of the technique used [221]. When combined with 4 mg of triamcinolone acetonide per 1 cm^2^, side effects such as necrosis and pain are minimal, although there may be an increased risk of dermal atrophy [221].

Intralesional verapamil (calcium channel blocker) injections are also used for keloid treatment. It has demonstrated the ability to reduce fibrous tissue production by stimulating procollagenase synthesis in keloids [224]. Research suggests that while monthly verapamil injections (2.5 mg/mL) may be safe, they might not be as effective as TAC (10 mg/mL) in preventing keloid recurrence after surgical excision [225]. However, a combination of verapamil with TAC injections showed promising results, significantly improved keloid scars, and long-lasting results [226]. Additionally, verapamil injections combined with surgical excision and core fillet flaps have proven to be a reliable and cost-effective method for treating earlobe keloids with a low recurrence rate [227]. Although the recurrence rate of keloids after verapamil treatment varies widely (1.4% to 48%), it remains a viable option, particularly when used in conjunction with other treatments like triamcinolone and surgery [225].

Intralesional botulinum toxin A has shown promise in keloid treatment due to its ability to decrease muscle tension, halt fibroblast cell cycles in the non-proliferative stage, and modulate TGF-β1 expression [228,229]. Studies have reported improved patient satisfaction and reduced erythema, pain, pliability, and itching following intralesional injections of botulinum toxin A [230,231]. A study showed that botulinum toxin A (70–140 units per session every three months) led to peripheral regression and lesion flattening without recurrence after one year [232]. Another RCT found that intralesional botulinum toxin A (5 IU/cm^3^ every eight weeks) significantly reduced keloid volume and height and softened lesions [233]. However, another study found no effect on keloid regression or fibroblast activity [229]. A double-blinded study revealed botulinum toxin A was not superior to corticosteroids in preventing keloid recurrence [234]. Despite mixed results, recent research highlighted botulinum toxin A’s adjuvant properties [235]. Combining it with intralesional TAC significantly improved pain and pruritus compared to TAC alone [235]. Botulinum toxin A combined with surgery also effectively treated post-otoplasty keloids [236]. A systematic review and meta-analysis of 15 RCTs concluded that intralesional botulinum toxin A was more effective for keloids than corticosteroids or placebo [237].

## 4. Novel and Emerging Therapies for Keloids

Emerging therapies focus on biologics targeting specific immune pathways, small molecule inhibitors, RNA interference, and gene therapy approaches (Table 2).

### 4.1. Biologics and Small Molecule Inhibitors

Biologics and small molecule inhibitors are emerging as promising therapeutic options for the treatment of keloid scars. These therapies target specific molecular pathways involved in the pathogenesis of keloids, aiming to disrupt the signaling processes that drive excessive fibroblast proliferation and collagen production.

One of the key targets for biologics in keloid treatment is the TGF-β/Smad signaling pathway. TGF-β1 and TGF-β2, which are involved in fibrosis and inflammation, are elevated in keloids, and specifically, TGF-β1 has been linked to increased collagen and fibronectin synthesis in keloids [241,242]. Fresolimumab, a monoclonal antibody that neutralizes all three TGF-β isoforms, has shown potential in reducing fibrosis in clinical trials for various fibrotic and cancer disorders [243]. In fibrotic diseases like systemic sclerosis, fresolimumab treatment has been shown to reduce biomarkers and improve clinical symptoms by decreasing the expression of TGF-β and collagen-related genes and inhibiting fibroblast infiltration [244]. Given the role of fibroblasts in keloid pathogenesis, fresolimumab may offer a novel therapeutic strategy for keloids.

Various inhibitors targeting the JAK-STAT pathway have also shown potential therapeutic effects on keloids. Ruxolitinib, a JAK1/2 inhibitor, blocks accelerated wound closure in keloid fibroblasts [245]. AG490, a JAK2 inhibitor, induces apoptosis and cell cycle arrest, reducing STAT3 phosphorylation and collagen production [97]. Other inhibitors like ASC-J9, Cucurbitacin I, STAT3 small interfering RNA (siRNA) and green tea polyphenol epigallocatechin gallate also suppress STAT3 signaling, decreasing collagen synthesis and fibroblast proliferation [90,91,246,247].

Additionally, small molecule inhibitors targeting the MAPK and PI3K/AKT pathways are under investigation for their potential to reduce keloid formation. Sorafenib has been shown to induce cell cycle arrest in keloid fibroblasts by inhibiting the TGF-β/Smad and MAPK/ERK signaling pathways [248]. Also, a recent study suggested that sunitinib effectively inhibits keloid development through suppression of the Akt/PI3K/mTOR pathway [115]. This study, using a keloid explant model in nude mice, demonstrated complete keloid regression after sunitinib treatment [115]. Sunitinib effectively inhibited keloid fibroblast proliferation, invasion, and collagen production while inducing apoptosis and cell cycle arrest [115]. These effects were associated with a marked inhibition of the PI3K/AKT/mTOR signaling pathway in keloid fibroblasts [115]. Also, CUDC-907, which is a PI3K/AKT/mammalian target of rapamycin (mTOR) pathway inhibitor, has demonstrated efficacy in proliferation, migration, invasion, and ECM deposition of KFs [116].

### 4.2. RNA Therapy

RNA interference (RNAi) is a cellular mechanism that utilizes small interfering RNAs (siRNAs), typically 21–22 nucleotides long, to silence specific gene expression through targeted mRNA degradation [248,249]. This approach has shown promise in keloid treatment by inhibiting gene expression in keloid fibroblasts. For instance, siRNA targeting TIMP-1/-2 has been observed to degrade collagen type I in keloid fibroblasts, while siRNA knockdown of heat shock protein 70 has resulted in significantly reduced collagen production [250,251]. Also, siRNA targeting the human wingless-related mouse mammary tumor virus integration site 2 has demonstrated the ability to slow down the growth and delay the cell doubling time of transfected keloid fibroblasts [252]. A recent study investigated the use of Runx2 siRNA to knockdown Runx2 mRNA in hypertrophic keloid fibroblasts (HKFs) and found that si-Runx2 transfection significantly inhibited the biological functions of HKFs, including proliferation, migration, and extracellular matrix deposition [110]. In another study, dissolvable hyaluronic acid (HA) microneedle patches loaded with siRNA for secreted protein acidic and cysteine-rich (SPARC) have effectively reduced collagen production and improved scar appearance and symptoms, demonstrating the practical benefits and efficacy of RNA-based treatments for pathological scars [253].

RNA therapeutics offer several advantages. They can target a wide range of genes, including those previously considered “undruggable”, and their high specificity can potentially reduce side effects compared to traditional small molecules [253,254]. Despite these advantages, challenges remain in the application of RNA therapeutics, such as effective delivery to target tissues, RNA instability, off-target effects, and potential immunogenicity [253,254,255,256].

### 4.3. Mesenchymal Stem Cell Therapy

Mesenchymal stem cell (MSC) therapy has garnered significant interest as a novel approach to treating keloid scars due to its anti-inflammatory and anti-fibrotic properties [257,258,259]. MSCs can be harvested from various sources, including bone marrow and adipose tissue, and expanded ex vivo under specific conditions to enhance their therapeutic potential [260,261]. These cells exhibit low immunogenicity, allowing for allogeneic transplantation [262].

MSC transplantation has been shown to effectively improve macroscopic and histological outcomes in keloid scars without significant complications [263]. MSCs exert their effects through the secretion of chemokines and microvesicles, which mediate the transition of macrophages from a pro-inflammatory M1 phenotype to an anti-inflammatory M2 phenotype, thereby attenuating inflammation and promoting tissue homeostasis [264,265,266].

Furthermore, MSCs have been found to reduce collagen deposition and contraction in scar tissues, which is a critical factor in the formation and persistence of keloid scars [267,268,269,270]. The potential of MSC-conditioned media, which contains bioactive extracellular vesicles, has also been highlighted as a promising cell-free therapeutic option [268,270,271,272]

However, there are no human clinical trials to date that have investigated the efficacy of MSC therapy for keloid scars. Future research needs to focus on identifying optimal MSC sources, standardizing delivery methods, and establishing reliable animal models to facilitate the translation of these findings to human applications.

## 5. Discussion and Conclusions

Keloid scars represent a significant clinical challenge due to their multifactorial nature and complex pathogenesis. They are characterized by dysregulated wound healing processes, chronic inflammation, and persistent fibroblast activation, leading to excessive extracellular matrix (ECM) production. Despite extensive research, the exact mechanisms underlying keloid formation remain incompletely understood, and effective treatments are limited. The involvement of key molecular pathways, including TGF-β/Smad, JAK/STAT, MAPK, and PI3K/AKT, plays a critical role in the development of keloids by promoting fibroblast proliferation, collagen synthesis, and ECM deposition.

The TGF-β/Smad pathway is central to keloid formation, enhancing fibroblast proliferation and collagen synthesis. Overexpression of TGF-β1 in keloid fibroblasts leads to increased ECM production and resistance to apoptosis. The JAK/STAT pathway, activated by pro-inflammatory cytokines, contributes to the dysregulated cellular responses observed in keloids, with STAT3 signaling particularly upregulated in keloid fibroblasts. The MAPK pathway interacts with the TGF-β/Smad pathway, influencing fibroblast proliferation and collagen accumulation. The PI3K/AKT pathway regulates fibroblast proliferation and differentiation into myofibroblasts, contributing to the fibrotic phenotype of keloids.

Current treatments for keloids, including silicone dressings, topical corticosteroids, cryotherapy, surgical excision, radiation therapy, laser therapies, and intralesional injections, aim to reduce scar size and symptoms but often result in high recurrence rates and potential side effects. Silicone dressings are effective in reducing scar thickness and color improvement through hydration and modulation of cell signaling. Topical corticosteroids reduce inflammation and fibroblast proliferation but require frequent application and monitoring for adverse effects. Cryotherapy reduces keloid volume and symptoms but is associated with hypopigmentation in darker skin types. Surgical options are effective for large keloids but have high recurrence rates if not combined with adjuvant therapies. Radiation therapy significantly reduces recurrence rates when used post-surgery but carries risks of skin atrophy and pigmentation changes. Laser therapies improve scar appearance and reduce symptoms, often requiring multiple sessions. Intralesional injections deliver therapeutic agents directly into keloid tissue, effectively flattening scars and alleviating symptoms.

Emerging therapies focus on targeting specific molecular pathways to disrupt the signaling processes driving keloid formation. Biologics and small molecule inhibitors target pathways such as TGF-β/Smad and STAT3 to reduce fibroblast proliferation and ECM production. RNA interference utilizes siRNAs to silence specific gene expression, showing promise in reducing collagen synthesis and fibroblast activity. Mesenchymal stem cell therapy exhibits anti-inflammatory and anti-fibrotic properties, potentially regenerating damaged tissue and modulating the immune response.

The multifactorial nature of keloid pathogenesis and the limitations of current treatments underscore the need for continued research and development of more effective therapies. While established treatments provide varying degrees of success, emerging therapies targeting specific molecular pathways offer promising new avenues for keloid management. Future research should focus on elucidating the precise mechanisms underlying keloid formation and translating these scientific discoveries into practical treatments to significantly improve the quality of life for individuals affected by keloid scars. By advancing our understanding of keloid pathogenesis and refining therapeutic strategies, we can move towards more effective and durable treatment outcomes, ultimately reducing the burden of keloid scars on patients.

## Figures and Tables

**Table 1:** Established treatments for keloid management.

Treatment	Mechanism of Action	Clinical Efficacy	Advantages	Potential Side Effects	References
Silicone Dressing	Create an occlusive barrier, maintain hydration of stratum corneum, reduce collagen production	Effective in reducing scar height, redness, and pruritus when used consistently	Non-invasive, easy to use, transparent, can be worn discreetly, suitable for various body parts	Minimal side effects, occasional skin irritation or maceration	[127,128,129,130]
Topical Corticosteroids	Penetrate skin, interact with glucocorticoid receptors, reduce production of inflammatory mediators	Effective in flattening keloid scars and reducing symptoms when used in conjunction with other therapies	Non-invasive, suitable for patients not candidates for aggressive treatments	Long-term use can lead to skin atrophy telangiectasia and requires careful monitoring	[131,132,133,134,135,136,137,138,139,140,141,142,143,144,145,146,147,148]
Cryotherapy	Freezes scar tissue, causing cell injury and ischemic necrosis, reducing keloid volume and symptoms	Reduces keloid scar volume by an average of 51% to 63%; scar recurrence rates range from 0% to 24%	Non-invasive, can be combined with other treatments	Hypopigmentation, particularly in darker skin types	[149,150,151,152,153,154,155,156,157]
Surgical Options	Remove keloid tissue, often combined with adjuvant therapies to minimize recurrence	Effective for large or resistant keloids, recurrence rates vary	Directly removes the bulk of keloid, which can improve the cosmetic appearance	High recurrence rates, if not combined with adjuvant therapies, potential for infection and scarring	[158,159,160,161,162,163,164,165,166,167,168,169,170,171,172,173,174]
Radiation Therapy	Inhibits fibroblast proliferation and collagen production, usually administered post-surgery	Reduces recurrence rates significantly when used as an adjuvant to surgery	Non-invasive, effective in combination with surgery	Temporary skin erythema and hyperpigmentation, rare long-term effects such as skin atrophy and telangiectasia	[175,176,177,178,179,180,181,182,183,184,185,186,187,188]
Laser Therapies	Target scar tissue with specific wavelengths of light, reduce size, color, and symptoms	Effective in improving appearance and reducing symptoms, especially when combined with other treatments	Non-invasive to minimally invasive, can be tailored to scar characteristics	Temporary redness, swelling, and changes in skin pigmentation; requires multiple sessions	[189,190,191,192,193,194,195]
Intralesional Injections	Deliver therapeutic agents directly into keloid tissue to reduce size and symptoms	Effective in flattening scars and alleviating symptoms, varies by agent	The targeted approach minimizes systemic side effects	Skin atrophy, hypopigmentation, telangiectasia with repeated corticosteroid injections	[196,197,198,199,200,201,202,203,204,205,206,207,208,209,210,211,212,213,214,215,216,217,218,219,220,221,222,223,224,225,226,227,228,229,230,231,232,233,234,235,236,237]

**Table 2:** Novel and emerging therapies for keloid management.

Therapy	Mechanism of Action	Advantages	Potential Side Effects	References
Biologics and Small Molecule Inhibitors	Target specific molecular pathways such as TGF-β/Smad and STAT3 to reduce fibroblast proliferation and ECM production	Targeted approach, potential to address underlying molecular causes	Varies by agent, the potential for immune reactions and off-target effects	[90,91,97,115,116,241,242,243,244,245,246,247,248]
RNA Therapy	Use siRNA to modulate gene expression involved in fibrosis	Specific and targeted modulation of gene expression, potential for long-term effects	Delivery challenges, potential for off-target effects and immune responses	[110,248,249,250,251,252,253,254,255,256]
Mesenchymal Stem Cell Therapy	Inhibit fibroblast proliferation and activity via paracrine signaling and immune modulation	Potential to regenerate damaged tissue, modulate immune response	Survival and engraftment challenges, long-term safety and efficacy not fully understood	[257,258,259,260,261,262,263,264,265,266,267,268,269,270,271,272]
